# Lung Cancer Stem Cell Lose Their Stemness Default State after Exposure to Microgravity

**DOI:** 10.1155/2014/470253

**Published:** 2014-09-07

**Authors:** Maria Elena Pisanu, Alessia Noto, Claudia De Vitis, Maria Grazia Masiello, Pierpaolo Coluccia, Sara Proietti, Maria Rosaria Giovagnoli, Alberto Ricci, Enrico Giarnieri, Alessandra Cucina, Gennaro Ciliberto, Mariano Bizzarri, Rita Mancini

**Affiliations:** ^1^Department of Experimental and Clinical Medicine, Magna Graecia University, 88100 Catanzaro, Italy; ^2^Department of Surgery “P.Valdoni,” Sapienza University, 00161 Rome, Italy; ^3^Department of Clinical and Molecular Medicine, Sapienza University, 00161 Rome, Italy; ^4^IRCCS Istituto Nazionale Tumori, Fondazione G. Pascale, 80131 Napoli, Italy; ^5^Azienda Ospedaliera S. Andrea, 00189 Rome, Italy; ^6^Department of Experimental Medicine, Sapienza University, 00161 Rome, Italy

## Abstract

Microgravity influences cell differentiation by modifying the morphogenetic field in which stem cells are embedded. Preliminary data showed indeed that stem cells are committed to selective differentiation when exposed to real or simulated microgravity. Our study provides evidence that a similar event occurs when cancer stem cells (CSCs) are cultured in microgravity. In the same time, a significant increase in apoptosis was recorded: those data point out that microgravity rescues CSCs from their relative quiescent state, inducing CSCs to lose their stemness features, as documented by the decrease in ALDH and the downregulation of both Nanog and Oct-4 genes. Those traits were stably acquired and preserved by CSCs when cells were placed again on a 1 g field. Studies conducted in microgravity on CSCs may improve our understanding of the fundamental role exerted by biophysical forces in cancer cell growth and function.

## 1. Introduction

Stem cells are highly responsive undifferentiated cells, embedded inside tissue's niches, able to adapt and differentiate into an appropriate cell type based on the microenvironment within which they reside. Growing evidence shows that physical microenvironments and mechanical stresses, independent of soluble factors, help influence stem-cell transition toward differentiated phenotypes [1]. Physical forces acting through the microenvironment may participate in providing positional information and nonlocal control on differentiating processes, displaying their effect at different system levels, from the cell to tissues [2]. Indeed, biophysical constraints participate in shaping the morphogenetic field, a construct that encapsulates key properties of instructive growth and patterning control [3]. A relevant, if generally underestimated, component of the morphogenetic field is represented by gravity, thought to act on cell behavior through both direct and indirect effects [4], the former exerted according to the nonequilibrium dynamics rules, as first theoretically suggested by Kondepudi and Prigogine [5]. Accordingly, a meaningful example of nonequilibrium reaction influenced by gravity in living systems is represented by microtubules and microfilaments dynamics, as well as by shape and phenotype differentiation [6, 7].

Recently, numerous studies have demonstrated the effects of microgravity upon stem cells. Rat mesenchymal stem cells cultured in simulated microgravity showed the strong proliferative characteristic of stem cells and retained their ability to differentiate into hyaline cartilage after transplantation [8]. Furthermore, human stem cells differentiate during space flight, and upregulation of genes related to various processes of neural development, neuron morphogenesis, and transmission of nerve impulse and synapse has been documented to occur during that process [9, 10]. Microgravity exposition induces different stem cell lineages to selectively differentiate into diverse phenotypes, yet that process is associated with conflicting results correlated to the proliferation rate: in fact, the proliferation capabilities of the different stem cell lines greatly differ among them [11–13], given that microgravity extensively changes the distribution of the cell cycle phase in mammalian stem cells [14].

To date, however, no investigations have been carried out on cancer stem cells (CSCs), a subset of cancer cells thought to play a pivotal role in tumor development as well as in tumor pharmacological resistance [15].

Stem cells are usually recognized on the basis of their functional and morphological features. A widely used approach which has been used for identification of stem cell subsets in vitro is sphere forming assays [16]. Many normal stem cells, such as hematopoietic or stem cells from tissues are capable, under special culture conditions, to form three-dimensional spheres, which can differentiate into multiple cell types. As with normal stem cells, CSCs isolated from brain or prostate tumors also have the ability to form anchorage-independent spheres [17, 18]. Additionally, for isolating CSCs from solid and haematological tumors, specific markers for normal stem cells of the same organ are commonly used. Among the putative markers for lung cancer, ALDH activity [19–24] and Nanog and Oct-4 mRNA expression [19] have been reported to be highly reliable parameters.

As previously reported, the stable non-small cell lung cancer (NSCLC) cell line H460 is enriched in cancer stem-like cells when grown in sphere medium forming 3D spheroids [25, 26]. The capability to generate spheroids correlated with increased ALDH activity, as well as with Nanog and Oct-4 mRNA expression [19, 20].

Herein, by using H460 spheroids generated in conventional culture condition as previously reported, we investigate the impact of simulated microgravity obtained by means of random positioning machine, on Lung CSCs. Despite some limitations, simulated weightlessness obtained by culturing cells in the random positioning machine (RPM) is a useful tool for microgravity-based cell study. Indeed, the RPM can support certain conditions of the space microgravity environment, including the lack of sedimentation to facilitate growth of multicellular spheroids [27, 28]. Moreover, the RPM can support the transition from 2D monolayer to 3D spheroid culture during continuous randomized rotation, and it has been suggested that the RPM may facilitate the study of cellular events that occur during this shift [29].

## 2. Materials and Methods

### 2.1. Cell Line

Established human non-small lung cancer cells (NSCLC) H460 was obtained from ATCC. Cells in adherent condition were cultured in RPMI-1640 (Sigma, St. Louis, MO, USA) and supplemented with 10% FBS (Sigma, St. Louis, MO, USA) at 37°C in an atmosphere of humidified air with 5% CO_2_. In the various experiments, reported cells were grown, treated, and analysed under identical conditions except for the absence or presence of microgravity.

### 2.2. Sphere Formation Assay

To determine the self-renewal ability, sphere propagation assay was performed as previously described [19]. Briefly, adherent lung cancer cells were suspended in serum-free DMEM/F12 (Sigma, St. Louis, MO, USA) containing insulin, glucose, heparin, bFGF, EGF (Sigma, St. Louis, MO, USA), B27 (Gibco, Invitrogen, Carlsbad, CA, USA), and plated in nonadherent culture to form spheres. After 48 h, individual spheres were formed, dissociated with accumax (Millipore, Temecula, CA, USA) and 20000 single cells/mL were used to obtain second generation of spheroids. Formation of individual spheres was observed after 24–48 h. Number of spheroids was measured or counted on an inverted microscope. An average of 8–10 fields was used for these measurements.

### 2.3. Microgravity Exposure and Cell Treatments

The cells are seeded at 20000/mL and the flasks were completely filled with DMEM/F12 supplemented with growth factors to avoid the presence of air bubble, capped, and transferred into a Desktop RPM, a particular kind of 3D clinostat [30], manufactured by Dutch Space (Leiden, the Netherlands). The degree of microgravity simulation depends on angular speed and on the inclination of the disk. These tools do not actually eliminate the gravity, but allow us to apply a stimulus rather than a unidirectional omnidirectional 1 g. Effects generated by the RPM are comparable to those of the real microgravity, provided that the direction changes are faster than the response time of the system to gravity field. The desktop RPM we used has been positioned within an incubator (for maintaining temperature, CO_2_, and humidity levels) and connected to the control console through standard electric cables. Before exposing the cells to the regime of simulated microgravity, the flasks were filled completely with fresh culture medium to eliminate the presence of air bubbles and, therefore, decrease the effects of turbulence and shear stress during rotation.

In the conditions employed in the experiments reported below, cells were exposed continuously in the RPM for 6–24–48 h. Similarly the control flasks filled with the same DMEM/F12 medium were cultured on ground (static) condition. After 6–24–48 h, the cells are harvested after accumax digestion and centrifugation at 1200 rpm and then collected for ALDH, cell cycle, and apoptosis analyses.

### 2.4. ALDH Analysis

ALDH activity was analyzed by the Aldefluor kit (Stem Cell Technologies, Vancouver, BC, Canada) according to manufacturer's instructions. Briefly, second generation of spheroids were dissociated and washed twice with Phosphate Buffered Saline (PBS) (Sigma, St. Louis, MO, USA), then the cells were incubated with ALDEFLUOR substrate (BODIPY-aminoacetaldehyde (BAAA)) in presence or not of specific ALDH inhibitor (diethylaminobenzaldehyde (DEAB)) for 30 minutes at 37°C. Cells suspended in ALDEFLUOR buffer together with BAAA and the DEAB were used to establish the baseline fluorescence and to define the ALDEFLUOR positive region. Cells that could catalyze BAAA to its fluorescent product BODIPY-aminoacetate (BAA) were considered ALDH positive.

### 2.5. Cell Cycle Analysis

Cells were collected and centrifuged and pellets were trypsinized and washed twice with PBS. Cells were fixed with 70% ethanol at 4°C for 24 h and stained with DNA PREP Stain (Beckman Coulter, Fullerton, USA) at 4°C overnight. Stained cells were measured by flow cytometry. Cell cycle analysis was performed in three independent experiments.

### 2.6. Apoptosis Analysis

*Annexin V/7-AAD Staining*. Second generation of spheroids was collected and centrifuged and pellets were trypsinized and washed twice with PBS. The cells were stained with FITC labeled annexin V/7-AAD (7-aminoactinomycine-D) according to the manufacturer's indication (annexin V/7-AAD kit; Beckman Coulter, Marseille, France). In particular, a washed cell pellet (5 × 10^4^ cells/mL) was resuspended in 500 *μ*L binding buffer; 10 *μ*L of annexin V together with 20 *μ*L 7-AAD was added to 470 *μ*L cell suspension. The cells were incubated for 15 min on ice in the dark. Apoptosis assay was performed three times.

ALDH, cell cycle, and apoptosis were evaluated by flow cytometry using an EPICS Coulter XL (Beckman-Coulter Inc.). Data were analyzed by Modfit LT Software (Veruty Software Inc., USA).

### 2.7. Real-Time PCR (RT-PCR) Analysis

For RT-PCR experiments, H460 spheroids were cultured under static or RPM condition for 6–24 and 48 h. Total RNA was isolated with Trizol Reagent (Life Technologies, Gaithersburg, MD, USA) according to the manufacturer's guidelines. RNA was digested with DNAase I (Invitrogen, Carlsbad, CA, USA) and reverse-transcribed into cDNA using High Capacity RNA-to cDNA Kit (Applied Biosystems, Life Technologies, Gaithersburg, MD, USA). Quantitative RT-PCR was performed using SYBR green detection (Applied Biosystem, Life Technologies, Gaithersburg, MD, USA) and the ∆∆Ct method for relative quantification. Expressions of Actin and GAPDH were used as internal controls.

The primers used for individual genes are indicated in Noto et al. [20].

### 2.8. Western Blotting Analysis

Cell lysates obtained using RIPA buffer (Sigma) were separated on SDS/PAGE acrylamide gel and transferred overnight on nitrocellulose membranes. Membranes were blocked with 5% milk and incubated overnight with the appropriate primary antibody, followed by the secondary antibody HRP-conjugated, and developed with ECL western blotting substrate (Promega, Madison, WI, USA). The primary antibodies used were the following: cyclin B1, cyclin D1 (Santa Cruz, Dallas, USA), and anti-vinculin (Sigma). All results were normalized over vinculin.

### 2.9. Optical Microscopy

Morphology of H460 was determined using optical images and cells were photographed with Nikon Coolpix 995 digital camera coupled with Zeiss Axiovert optical microscope. The images were obtained with a 100x and 320x magnification.

### 2.10. Statistical Analyses

Data were expressed as mean ± standard deviation (SD). Statistical comparisons were performed using Student's *t*-test. *P* values < 0.05 were considered statistically significant.

## 3. Results 

### 3.1. Microgravity Induces Changes in Spheroids-Forming Efficiency in H460 Cells

**Figure 1:**
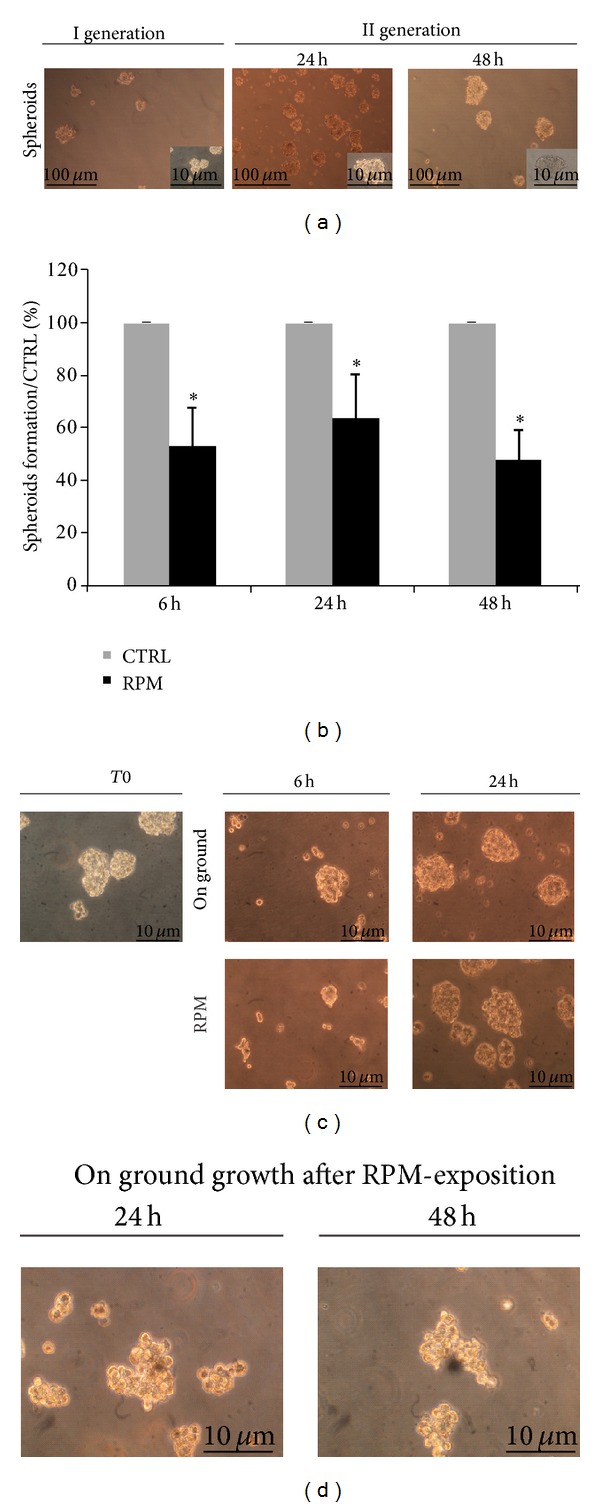
(a) Formation of H460 spheroids cultured in nonadherent conditions at the first and at the second generation after 24–48 h of seeding. (b) Spheroids-forming assay was carried out at 6–24 and 48 h of RPM-exposure and spheroids were counted by optical microscopy. Results represent mean ± SD (*n* = 3). Student's *t*-test, *P < 0.0.5. (c) Representative spheroids obtained from H460 cells on ground or RPM condition at 0–6 and 24 h. (d) H460 spheroids exposed to RPM and then cultured on ground for 24 and 48 h. H460 spheroids give rise to de novo irregular cell aggregates.

Cancer stem cells are morphologically identified because of their ability to grow as 3D nonadherent structures when clonally seeded in sphere medium. To investigate spheroids-forming efficiency, sphere propagation assay was carried out on H460 by resuspending single cells into DMEM/F12 supplemented with growth factors.  Figure 1(a) (left panel) shows typical spheroids obtained after 24–48 h of seeding (first generation). After 48 h, individual spheres were formed and dissociated and single cells were used to obtain a second generation of spheroids, as reported in Figure 1(a) (right panel).

In order to analyze CSCs self-renewal capability in a simulated microgravity field, H460 single cells derived from dissociation of spheroids of first generation were transferred to RPM and allowed to grow at 6–24 and 48 h. Results obtained showed a dramatic reduction in the number of spheroids developed in RPM cultures: indeed, RPM-treated cells showed a decrease of spheroids-forming efficiency to 53.1 ± 15.0%, 63.5 ± 17.3%, and 47.7 ± 11.7% at 6, 24, and 48 h, respectively (Figure 1(b)). Hence, we evaluated the morphology of spheroids growing in simulated microgravity. As shown in Figure 1(c), RPM-treated cells exhibited strong changes in the morphology of cell aggregates. Indeed, H460 formed well-rounded spheroids when growing on ground condition, whereas when exposed to a simulated microgravity field, cultures were characterized by poorly compact aggregates with scarcely delineated borders at 6 and 24 h (Figure 1(c)).

Interestingly, H460 cells exposed to RPM and then left to grow de novo on ground did not recover the capability to form spheroids (Figure 1(d)). Those results indicate that H460 cells mislay the morphological qualities of stemness during as well as after exposition to a microgravity field.

### 3.2. Effects of RPM on Cell Cycle and Apoptosis

It is widely recognized that microgravity exposition lead to impressive modification in cell cycle distribution [14]. Indeed, we observed in RPM-treated CSCs a significant redistribution of cells in between the different phases, after 24 hours of microgravity exposition. Cell cycle was investigated by using flow cytometry.

**Figure 2:**
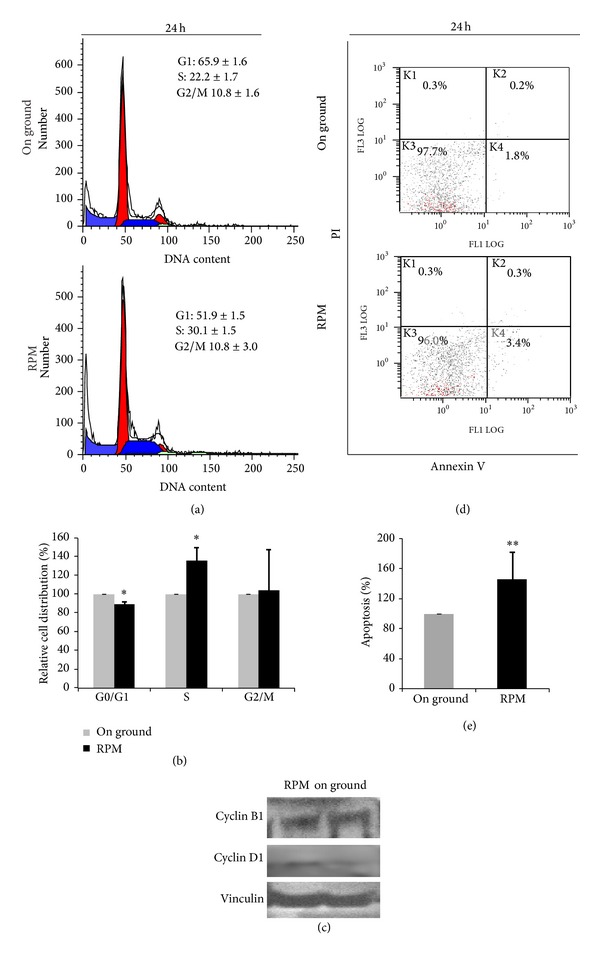
(a) A representative experiment of DNA content analysis by flow cytometry. (b) Quantitative analysis of DNA content after 24 h on ground or RPM exposure showing that RPM evokes shift from G0/G1 phase (Student's *t*-test, *P* = 0.04), into the S phase (Student's *t*-test, *P* = 0.01). The data shown represent the mean ± SD (*n* = 3). (c) A representative WB analysis of cyclin D1 and cyclin B1 in H460 spheroids exposed or not to RPM. Vinculin was used as loading control. (d) Apoptosis of H460 spheroids growing on ground and in simulated gravity. Graphs are representative of three independent experiments. (e) Apoptosis rate in CSCs growing on ground and in RPM-condition. Data represent mean ± SD (Student's *t*-test, *P* ≤ 0.01).

As shown in Figures 2(a)-2(b), cell cycle distribution significantly differed from on ground and RPM-treated CSCs: indeed, H460 spheroids exposed to simulated microgravity showed a relevant increase in S-phase distribution (+35.7 ± 14.6% *P* = 0.04), whereas a significant decrease in the percentage of cells in the G0/G1 phase (−11.4 ± 2.2% *P* = 0.01) was observed, when compared to control sample. Moreover, preliminary results showed an increase of cyclin D1 after 24 h of RPM exposure consistent with cell cycle progression and proliferation (Figure 2(c)).

Furthermore, microgravity induced a dramatic increase in apoptotic rate on CSCs cultured on RPM. After 24 h of RPM-exposure, apoptosis rate grew up to 145.95 ± 37.2% (*P* ≤ 0.01), when compared to on ground cultured samples (Figures 2(d)-2(e)).

### 3.3. RPM Reduces Stemness Quality of H460 Spheroids

**Figure 3:**
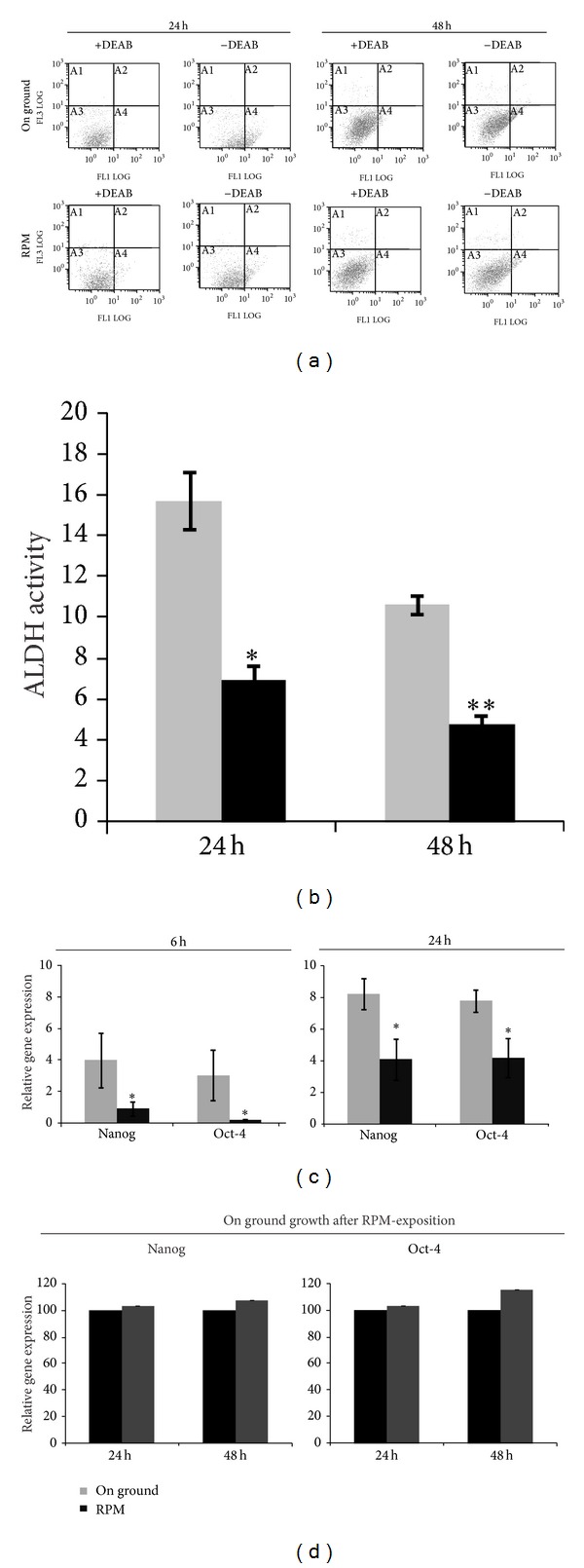
(a) ALDH assay performed on H460 cells by flow cytometry. The H460 were cultured in sphere medium and exposed to RPM for 24 and 48 h. Baseline fluorescence was established by inhibiting ALDH activity (with DEAB) (left) and used to identify ALDH positive cells (without DEAB). (b) The histogram shows the quantitative results obtained in three independent experiments. Data indicated a significant reduction of ALDH activity in H460 RPM-treated cells at 24 h (Student's *t*-test, *P* ≤ 0.02) and at 48 h (Student's *t*-test, *P* ≤ 0.01). (c) Evaluation of the relative expression of Oct-4 and Nanog by quantitative Real-Time PCR at 6 and 24 h. The results show a significant decrease of Nanog and Oct-4 in RPM-exposed cells at 6 h (Student's *t*-test, *P* ≤ 0.04) and at 24 h (Student's *t*-test, *P* ≤ 0.03). The data represent mean ± SD of three independent experiments. (d) Histograms represent the Oct-4 and Nanog mRNA expression in RPM-treated spheroids and in the same sample further exposed to normal gravity condition. The values were compared to these obtained after 24 h of RPM exposure. Preliminary data did not show strong differences between RPM and on ground growth after RPM-exposition.

We studied whether RPM could influence the differentiation state of H460 spheroids measuring ALDH activity.  Figure 3(a) shows representative data of ALDH activity analyzed by flow cytometry. Significant differences in the ratio of ALDH positive cells were observed between cells grown on RPM or on ground. Indeed, RPM exposure induced a significant decrease in ALDH activity, respectively, of 44.8 ± 8.4% (at 24 h, *P* ≤ 0.02) and 45.3 ± 5.6% (at 48 h, *P* ≤ 0.01), as shown in Figure 3(b).

In order to confirm these results, we measured the effects of RPM on the expression of Oct-4 and Nanog genes (stemness biomarkers). Quantitative Real-Time PCR on these markers revealed a significant lower Nanog and Oct-4 mRNA expression in RPM-treated cells at 6 and 24 h when compared with control (Figure 3(c)). Overall, those data suggest that RPM simulated microgravity induces the differentiation of H460 spheroids.

In order to verify if this effect could be considered a transitory consequence of microgravity, we have cultured on ground H460 cells previously exposed to RPM. Preliminary gene expression data showed no significant differences in Oct-4 and Nanog levels in between cells exposed to RPM and cells cultured on ground after 24 h of RPM treatment, suggesting, therefore, that the loss of stemness features was still maintained when H460 cells were reseeded in normal gravity (Figure 3(d)).

## 4. Discussion

Morphogenesis relies on a complex set of deep-rooted processes, involving selective proliferation, increased apoptosis, and phenotype differentiation [31]. Microgravity has been proven to foster those processes in different experimental models [32, 33]. Stem cells should, therefore, undergo several adaptive changes and mutually entrenched proliferation/apoptosis cycles to be eventually committed toward differentiation. To an end of that process, stem cells lose the “stemness default state,” as shown by the emergence of new morphologies and the downregulation of specific molecular markers. A suggestive body of literature has demonstrated that both simulated microgravity and weightlessness experienced during spaceflights can efficiently foster stem cell differentiation [9, 10]. Our study provides evidence that a similar event occurs even when cancer stem cells are exposed to microgravity. Lung CSCs were arrested in S phase as early as after 24 hours of microgravity. In the same time, a significant increase in apoptosis was recorded in CSCs growing in RPM. Overall, those data point out that microgravity rescues CSCs from their relative “quiescent” state, thereby conferring on them a dynamical profile that may eventually culminate in differentiation. Indeed, lung CSCs lose their stemness features, as documented by the decrease in ALDH and the downregulation of both Nanog and Oct-4 genes. That process is well manifested at both 24 and 48 hours of microgravity exposition. Unexpectedly, those traits were stably acquired by CSCs, given that, by reseeding on ground CSCs previously exposed to RPM, This does not induce don't show a phenotypic reversion. Therefore, it is unlikely that the differentiating commitment induced by microgravity could be a transient effect, although additional studies are needed to ascertain if CSCs entirely lose their malignant features. That result is quite surprising since we are dealing with cancer stem cells, which are supposed to be irreversibly oriented toward malignant transformation due to overwhelming signals provided by mutated genes: the possibility of reprogramming CSCs into differentiated cells through biophysical cues discloses indeed new opportunities for cancer understanding and treatment. Previous studies have already suggested that biophysical constraints may be effective in inducing phenotypic cancer reversion, by modifying the tensional balance as well as the cytoskeleton architecture [34]. Controlled studies conducted in microgravity can further expand our understanding of the fundamental role of biophysical forces in cancer cell growth and function and serve as a novel paradigm for innovation [35].
